# The underlying mechanism and targeted therapy strategy of miRNAs cross-regulating EMT process through multiple signaling pathways in hepatocellular carcinoma

**DOI:** 10.3389/fmolb.2024.1378386

**Published:** 2024-03-22

**Authors:** Juan Chen, Fuguo He, Hong Peng, Jinjun Guo

**Affiliations:** ^1^ Department of Pathology, Bishan Hospital of Chongqing Medical University, Chongqing, China; ^2^ Department of Gastroenterology, Bishan Hospital of Chongqing Medical University, Chongqing, China

**Keywords:** HCC, miRNA, EMT, signaling pathway, targeted therapy

## Abstract

The consistent notion holds that hepatocellular carcinoma (HCC) initiation, progression, and clinical treatment failure treatment failure are affected by the accumulation of various genetic and epigenetic alterations. MicroRNAs (miRNAs) play an irreplaceable role in a variety of physiological and pathological states. meanwhile, epithelial-mesenchymal transition (EMT) is a crucial biological process that controls the development of HCC. miRNAs regulate the intermediation state of EMTor mesenchymal-epithelial transition (MTE)thereby regulating HCC progression. Notably, miRNAs regulate key HCC-related molecular pathways, including the Wnt/β-catenin pathway, PTEN/PI3K/AKT pathway, TGF-β pathway, and RAS/MAPK pathway. Therefore, we comprehensively reviewed how miRNAs produce EMT effects by multiple signaling pathways and their potential significance in the pathogenesis and treatment response of HCC. emphasizing their molecular pathways and progression in HCC initiation. Additionally, we also pay attention to regulatory mechanisms that are partially independent of signaling pathways. Finally, we summarize and propose miRNA-targeted therapy and diagnosis and defense strategies forHCC. The identification of the mechanism leading to the activation of EMT programs during HCC disease processes also provides a new protocol for the plasticity of distinct cellular phenotypes and possible therapeutic interventions. Consequently, we summarize the latest progress in this direction, with a promising path for further insight into this fast-moving field.

## 1 Introduction

Hepatocellular carcinoma is a malignant tumor derived from hepatocytes and hepatobiliary cells. The latest data show that liver cancer ranks sixth in new cases and third in mortality worldwide (Sung et al., 2021). In recent years, although great progress has been made on HCC molecular mechanism and therapy techniques, the prognosis of HCC patients is generally poor due to high metastasis and recurrence, which makes us need to examine this urgent problem from a broader perspective (Avila et al., 2006). Studies have shown chronic alcohol consumption or aflatoxin intoxication, chronic hepatitis B or C virus (HBV, HCV, *etc.*) infection or a high-fat diet that leads to obesity are clear etiological factors that contribute to the development of HCC for decades (Forner et al., 2012). The formation of HCC is a heterogeneous formation of hepatocytes and progenitor cells, both of which are epithelial cell types, and the plastic changes of the latter lead to the generation of EMT. At the same time, the production of EMT in hepatoma cells is systematically regulated by miRNAs (Gao et al., 2014). MicroRNA (miRNA) is a class of endogenous small non-coding RNA of 18–25 nucleotides long. It is well-recognized that miRNAs play a crucial role in the initiation and proliferation of various types of cancer through the regulation of dissimilar signaling pathways (Doghish et al., 2022). EMT endows cells with migration and invasion properties, induces stem cell properties, effectively prevents apoptosis and senescence, and contributes to immunosuppression. The mesenchymal state of cells is closely related to the ability of cells to migrate to distant organs. At the same time, the induced stemness of cells allows their subsequent differentiation into multiple cell types during development and the onset of metastasis (Thiery et al., 2009). Meanwhile, EMT is a key intermediate process that drives tumor metastasis. Accumulating evidence suggests that dysregulation of some microRNAs is involved in this process. more clearly speaking, ample evidence exists for the important regulatory potential of other miRNAs in conditions associated with TMT processes in HCC (Szabo and Bala, 2013). Notably, the precise regulation of HCC progression by EMT-mediated miRNAs needs to be better summarized and considered despite numerous efforts by researchers (Chen et al., 2016).

## 2 Classical miRNA production pathways

**FIGURE 1:**
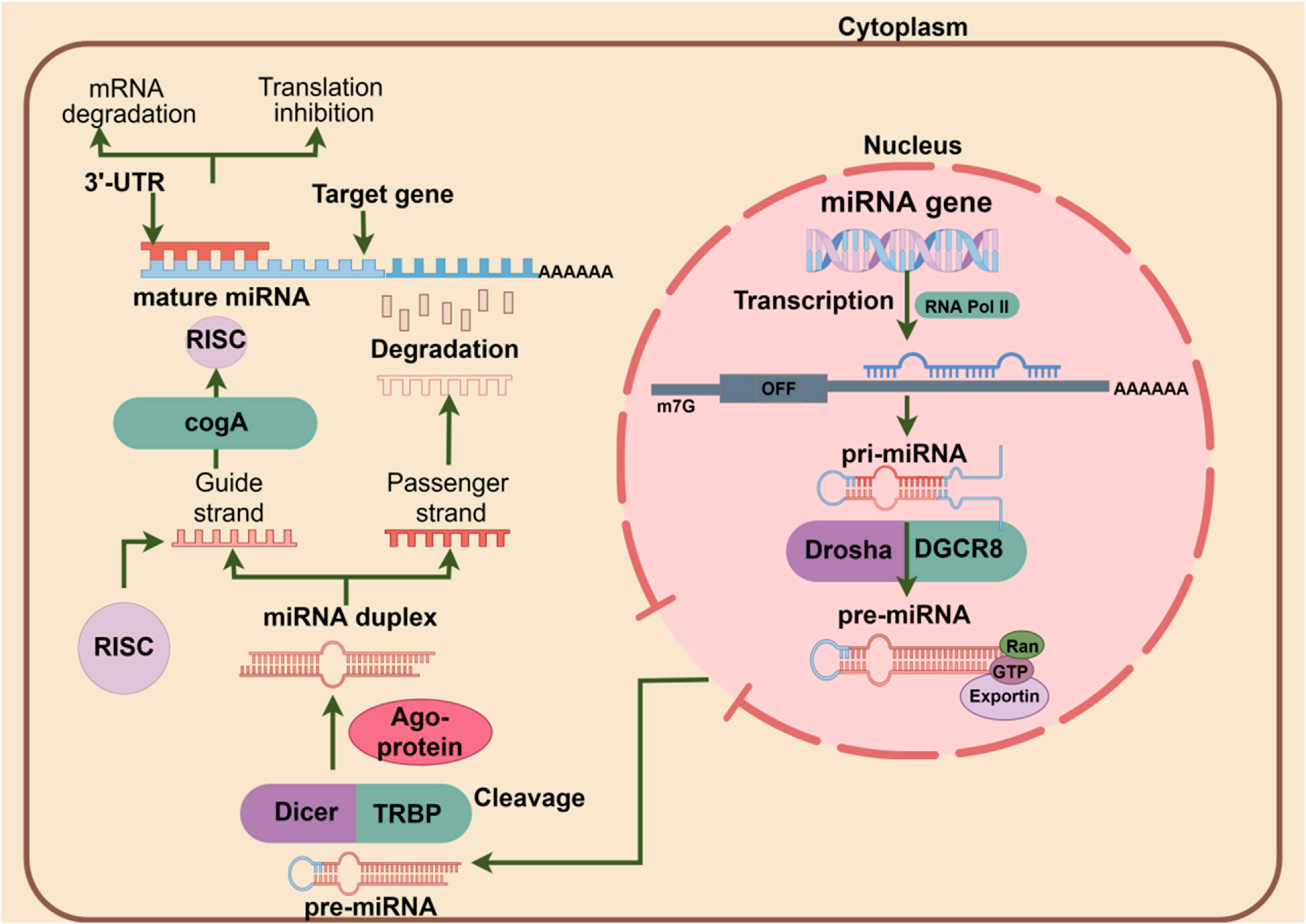
The pri-miRNAs in the nucleus are synthesized by RNA polymerase II. After processing by the microprocessor, it is transported to the cytoplasm and then treated by DICER. Finally, mature miRNAs are loaded onto AGO proteins and bind to target genes to exert their biological functions.

As integral parts of small regulatory RNAs, miRNAs do not encode proteins but play a critical role in post-transcriptional regulation by targeting the 3′-UTR of target gene transcripts to reduce the expression of homologous proteins and regulate cellular biological processes (Feng et al., 2022). The formation of miRNA is accompanied by four processes. When nuclear RNA polymerase II transcribes pri-miRNA with a hairpin structure (Lee et al., 2004), it is cleaved by the microprocessor to form pre-miRNAand then exported from the nucleus to the cytoplasm by Exportin five and RAN-GTP (Monica Ballarino et al., 2009). In the cytoplasm, pre-miRNA is further converted into miRNAs* by Dicer, and with the help of the chaperones HSC70/HSP90, the formed miRNA* is loaded into the Argonaute protein to form a silencing complex, whereby targeting and regulating the target gene (Iwasaki et al., 2010). Mechanistically, the silencing complex forms 3′-end extensive pairing on the one hand and then cleaves mRNA in animal cells through endonuclease properties (Bartel, 2018). On the other hand, the eukaryotic translation initiation factor 4E (eIF4E) transporter (4E-T) forms a translation barrier by recruiting DDX6 to bind the CCR4-NOT complex (Kamenska et al., 2014; Kamenska et al., 2016), preventing miRNA translation initiation and recruiting TNRC6 and tail-related regions through the Argonaute protein (PABPC) binding accelerates the rapid degradation of miRNAs (Jonas and Izaurralde, 2015). The schematic diagram of the canonical miRNA production and action mechanism is shown in Figure 1.

## 3 EMT

The concept of EMT was first proposed by Betty Hay in 1963 and described its pivotal role in embryonic development (Trelstad et al., 1967). EMT is mainly divided into three types according to the biological background of occurrence. Type I EMT occurs during embryonic development, and type II and type III EMT occurs during wound healing, tissue regeneration, and progression of cancer respectively (Lamouille et al., 2014). Notably, type III EMT is always accompanied by systemic metastasis of liver cancer cells, including lymph nodes, lungs, kidneys, and spleens. The characteristics of EMT during cancer metastasis is the suppression of the expression of the epithelial gene E-cadherin, resulting in the typical polygonal, cobblestone-like shape of epithelial cells with intercellular adhesions such as cell junctions, including tight junctions, adherens junctions, desmosomes, and gaps the junction gradually disintegrates and is accompanied by a loss of cell polarity. In addition, cells acquire a spindle-shaped mesenchymal morphology and express markers associated with the mesenchymal cell state, in particular N-cadherin, matrix metallopeptidase family proteins (MMPs), vimentin, and fibronectin (Trelstad et al., 1967; Lamouille et al., 2014; Dongre and Weinberg, 2018).

## 4 Multiple signaling pathways involved in EMT-mediated miRNA regulation of HCC

To shed light on a portion of the molecular underpinnings of miRNA regulating the EMT in cells, we, at least in part, introduce the interplay of multiple signaling pathways during HCC metastasis. Correspondingly, Studies have shown that EMT progression is regulated by a variety of signaling pathways. In addition to the common signaling pathway that promotes transforming growth factor-β (TGFβ) fibrosis through SMAD proteins, it can also activate PI3K-AKT, RAS/MAPK, and p38 MAPK and other key signaling pathways in HCC (Derynck, 2005; Xu et al., 2009). Phosphorylation of TβRI creates a site between growth factor receptor binding protein 2 (GRB2) and Sevenless (SOS) and initiates the RAS-RAF-MEK-ERK-MAPK pathway, where TGFβ-induced p38 MAPK (Lee et al., 2007). Some growth factors induce EMT through receptor tyrosine kinases (RTKs), including epidermal growth factor (EGF), fibroblast growth factor (FGF), hepatocyte growth factor (HGF), and vascular endothelial growth factor (VEGF). Furthermore, activated ERK1 and ERK2, which initiate MAPK, promote EMT by increasing the expression of EMT transcription factors and regulators of cell motility and invasion (Stoker, 1985; Rivera-Feliciano et al., 2006; Wanami et al., 2008; Shirakihara et al., 2011). Meanwhile, WNT/β-catenin signaling promotes EMT by inhibiting glycogen synthase. Glycogen synthase kinase-3 (GSK3β) is found to stabilize β-catenin, which translocates to the nucleus to engage the transcription factors lymphoid enhancer-binding factor 1 (LEF) and T cell factor (TCF) and promote EMT-friendly Gene Expression Program (Yang et al., 2006; Nawshad et al., 2007). Hypoxia in the tumor environment can also promote EMT through hypoxia-inducible factor 1α (HIF1α), thereby activating the expression of TWIST (Yang et al., 2008). Notably, the intervention of exosomes makes the interaction between various signaling pathways more effective, and the remote regulation of HCC by miRNAs through exosomes has also received further attention.

### 4.1 miRNAs regulate the EMT process of hepatoma cells via the Wnt/β-catenin signaling pathway

**FIGURE 2:**
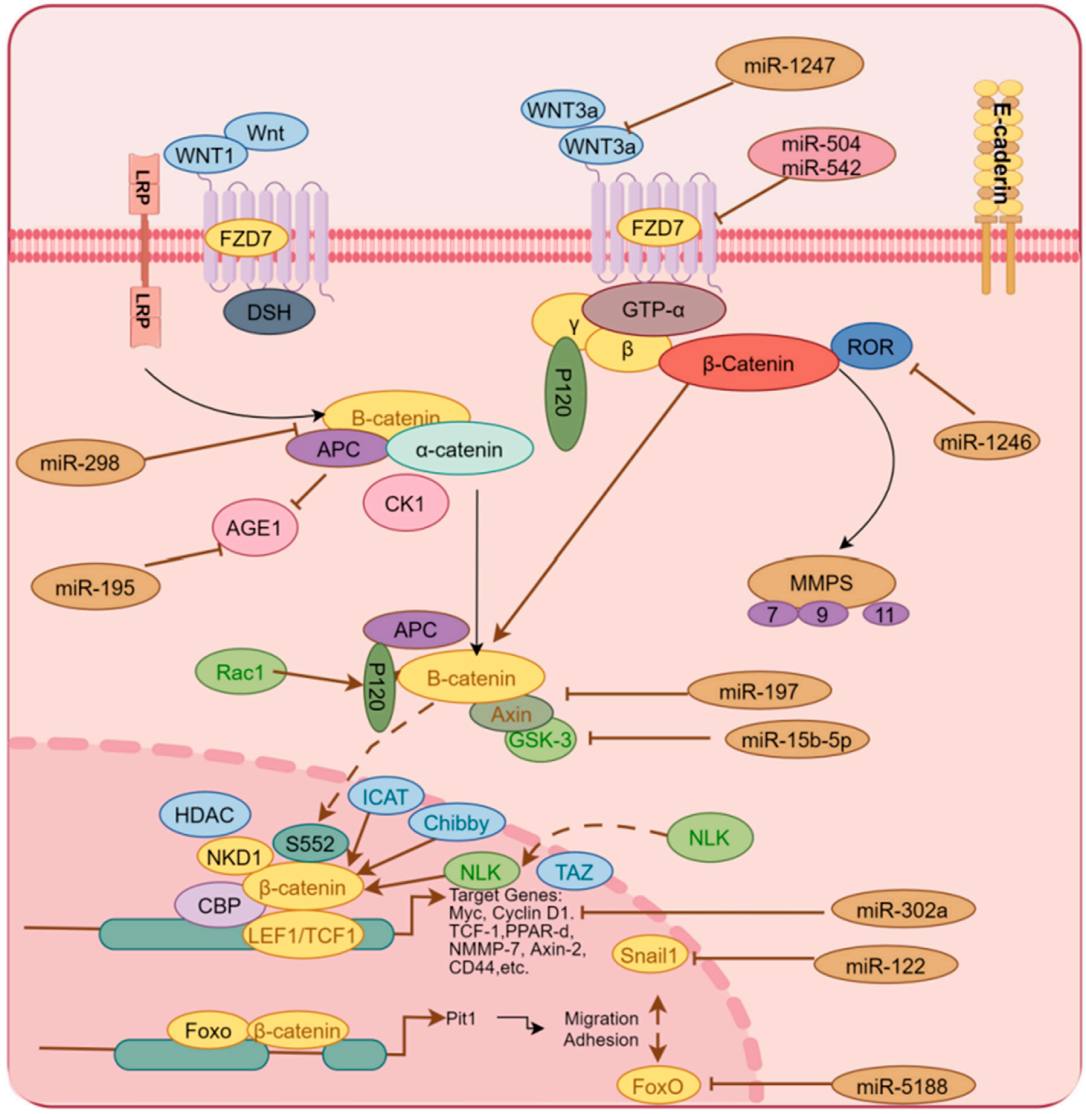
Multiple miRNAs regulate the activity of the Wnt/β-catenin signaling pathway by targeting some genes in the Wnt/β-catenin signaling pathway, including Wnt3 a, FDZ7, Axin, β-catenin and other proteins, thereby activating or blocking the EMT process of liver cancer cells.

High expression of *miR-5188* in HCC patients predicts poor prognosis. Existing evidence indicates that *miR-5188* directly targets FOXO1, which interacts with β-catenin in the cytoplasm to reduce nuclear translocation of β-catenin and promote Wnt signaling and downstream tumor activation EMT. The highly expressed miR-5188 inhibited the expression of FOXO1, promoted the accumulation of β-catenin in the nucleus, and activated the expression of interstitial genes to promote cell proliferation and invasion (Lin et al., 2019). High levels of *miR-197* expression promote EMT and invasion of HCC cells *in vitro* and *in vivo*, and mechanistically, *miR-197* directly targets Wnt/β-catenin signaling negative regulators Axin-2, Naked cuticle 1 (NKD1) and Dickkopf-related protein 2 (DKK2) thereby increasing the activity of TCF/LEF and accelerating the nuclear accumulation of β-catenin (Hu et al., 2018). Available data show that in clinical liver cancer patient samples and liver cancer cells, *miR-498* is significantly lower than that in normal patients and liver cells. The highly expressed *miR-498* can inhibit the proliferation, migration, and invasion of hepatoma cells by inhibiting ZEB2 (Zhang T. et al., 2018). Frizzled Class Receptor 7(FZD7), a co-receptor of Wnt signaling, is a key factor in the regulation of Wnt. Studies have shown that *miR-542-3p* has been demonstrated as a tumor suppressor in many cancers, inhibiting the proliferation and invasion of HCC by inhibiting the activation of the Wnt signaling pathway through targeted regulation FZD7 in liver cancer cells (Wu et al., 2017). Hepatic stellate cells trigger *miR-1246*, which targets binding to RORα and impairs the interaction of RORα protein with β-catenin, resulting in increased nuclear levels of β-catenin and decreased cytoplasmic β-catenin levels *in vitro* and *in vivo* (Huang et al., 2020). Similarly, *miR-504* targets FZD7 and β-catenin, and restoration of FZD7 expression significantly reversed the inhibitory effects of *miR-504* on HCC cell proliferation, invasion, and Wnt/β-catenin signaling (Quan et al., 2018). *miR-298* directly targets catenin delta 1 (CTNND1), and the low expression of *miR-298* may be a powerful assistant for the proliferation and invasion of liver cancer cells. Conversely, miR-298 overexpression and CTNND1 knockdown inhibited Wnt/β-catenin signaling and resulted in decreased expression of β-catenin, WNT11, Cyclin D1, and MMP7 in HCCLM3 cells, while inhibiting EMT (Cao N. et al., 2018). *miR-708* directly targets ZEB1 *in vitro*, which is involved in HSCs by regulating the Wnt/β-catenin signaling pathway, and highly expressed *miR-708* reverses EMT (Yang B. et al., 2020). *miR-122* expression is closely related to tumor size, vascular invasion, and patient survival in HCC patients. Mechanistic studies reveal that *miR-122* overexpression inhibits the EMT process by targeting Snail1 and Snail2, and regulates their expression levels in HCC cells, while the upregulated expression of *miR-122* inhibited the Wnt/β-catenin signaling pathway and slowed the EMT process of tumors (Jin et al., 2017). CircRNA mitochondrial tRNA translation optimization 1 (circMTO1) acts as a cavernous body of *miR-541-5p* to co-regulate HCC cell proliferation, migration, and invasion. The study found that upregulation of *miR-541-5p* inhibited the expression of ZIC1, promoted HCC cell proliferation, migration, and invasion, and inhibited cell apoptosis. In addition, the latter activated Wnt/β-catenin signaling to promote EMT. Silencing CircMTO1 could upregulate Wnt/β-catenin pathway markers, β-catenin, cyclin D1, c-myc and mesenchymal markers N-cadherin, Vimentin, and MMP2 downstream ZIC1 regulator expression, while the epithelial marker E-cadherin expression was downregulated (Li D.et al., 2021). Drug-induced, dependent on the Wnt/βcatenin signaling pathway, *miR-219-5p* targeted inhibition of glypican-3 (GPC3) inhibited HCC cell proliferation, migration, and invasion (Gong et al., 2019). *miR-194* targets the protein regulator of cytokinesis 1 (PRC1) to inhibit EMT, growth, proliferation, invasion, and migration in HCC cells *in vitro*, and mouse xenograft growth *in vivo*, which is Wnt/-dependent by biosignature analysis inactivation of catenin signaling (Zhang et al., 2019). As shown in Figure 2, miRNAs regulate Wnt/β-catenin by targeting multiple genes.

### 4.2 miRNA-dependent PTEN/PI3K/AKT signaling pathway regulates EMT process in HCC

Previous experimental data showed that *miR-25-3p*, *miR-130b-3p*, and *miR-425-5p* were transferred to macrophages via exosomes to induce M2 polarization in macrophages through CXCL12/CXCR4-mediated activation of the PI3K/Akt signaling pathway to regulate PTEN. The latter enhances EMT and secretes vascular endothelial growth factor (VEGF) to promote liver cancer cell metastasis (Wang et al., 2020). It has been reported that the vast majority of HCC is caused by liver fibrosis or cirrhosis (Bruix et al., 2014), miRNAs have attracted much attention in regulating hepatic fibrosis and promoting the metastasis of hepatocytes to cancer. We found that *miRNA-21* is associated with greater activation of cancer-associated fibroblasts (CAFs) and higher blood vessel density in HCC patients, as HCC cells secrete exosomes *miRNA-21* that directly targets PTEN, resulting in hepatic stellate cells (HSCs) Activation of PDK1/AKT signaling pathway. Activated CAFs further promote cancer progression by secreting angiogenic cytokines, including VEGF, MMP2, MMP9, b-FGF, and TGF-β, and enhance EMT progression in hepatoma cells (Zhou et al., 2018). *miR-425-5p* targets both SCAI and PTEN and promotes HCC cell invasion and metastasis through SCAI-mediated dysregulation of integrin β1-Fak/Src-RhoA/CDC42, PTEN-AKT, and TIMP2-MMP2/MMP9 signaling, clinical experimental results also showed that high expression of *miR-425-5p* also predicted poor prognosis and low long-term survival after surgery (Fang et al., 2017). Previous studies suggest that *miR-345* indirectly regulates transcription factors Slug, Snail, and Twist by targeting interferon regulatory factor 1 (IRF1), and activates the mTOR/STAT3/Akt signaling pathway, thereby slowing the EMT process of liver cancer (Yu Q. et al., 2017). Targeting tyrosine kinase receptor factor (Met) by *miR-148a* reduces Snail accumulation in the nucleus, promotes hepatocyte growth, activates downstream Akt Ser473 phosphorylation, and inhibits GSK-3β-Ser9 phosphorylation, thereby alleviating EMT cancer in the liver (Zhang et al., 2014).

Numerous pieces of evidence show that the malignant potential of HCC is regulated by the tumor microenvironment (TME) (Berindan-Neagoe and Calin, 2014; Steinbichler et al., 2017). This is partly owing to cancer-associated fibroblasts (CAFs) helping regulate tumor progression. Thus, understanding how they function in HCC could improve patient outcomes. In addition, another team found that exosomal *miR-21* and *miR-10b* induced by the acidic microenvironment of HCC promoted the proliferation and metastasis of cancer cells, and also triggered the activation of HIF-1α and HIF-2α to exacerbate the metastasis of HCC cells and ectopic colonization. Mechanistically, HIF-1α and HIF-2α can bind to the promoter HRE sites of *miR-21* and *miR-10b*, activate the overexpression of *miR-21* and *miR-10b* to inhibit the tumor suppressor PTEN and activate p-AKT/mTOR signaling pathway, thereby inhibiting epithelial gene expression, promoting mesenchymal gene synthesis, and ultimately accelerating the EMT process (Tian et al., 2019). Studies have shown that *miR-150-3p* inhibits HCC migration and invasion through exosomes. Although the specific mechanism has not been well explained, however, The notion that low expression of *miR-150-3p* in HCC tissue is an important factor in the recurrence of HCC patients is well supported by clinical data (Yugawa et al., 2021). In recent years, the regulation of HCC progression by exosomal *miR-155-5p* has attracted the attention of researchers. *miR-155-5p* activates the PI3K/Akt pathway in HCC by directly targeting the 3′-UTR of the tumor suppressor PTEN to promote proliferation, Invasion, and migration, inhibiting apoptosis (Fu et al., 2017; Sun et al., 2019). Interestingly, both *miRNA-32-5p* and *miR-92a-3p* have the same mechanism of action as *miR-155-5p* in the hepatocyte EMT process and enhance the resistance of hepatoma cells to multiple drugs (Fu et al., 2018; Yang J. et al., 2020).

**FIGURE 3:**
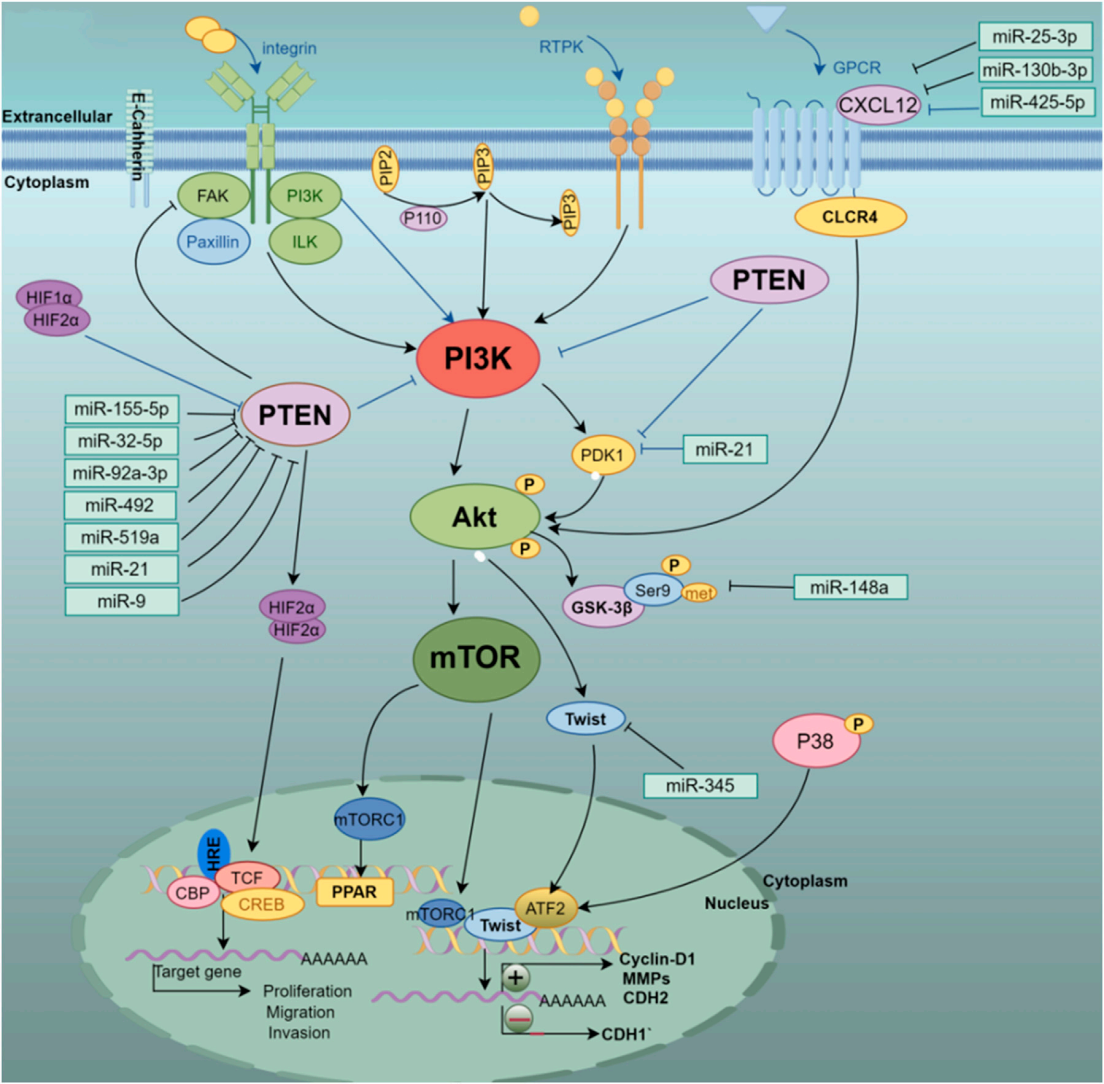
Multi-miRNA targeted regulation of PTEN/PI3K/AKT pathway-related genes, thereby activating or inhibiting the PI3K/Akt/mTOR signaling pathway to affect the occurrence and development of EMT in HCC cells.

In addition, the evidence provides a clear view that *has-miR-181a* promotes liver metastasis in colorectal cancer. The study found that *has-miR-181a* promotes cell proliferation and invasion by combining with the tumor suppressor WIF-1. On the contrary, inhibiting the expression of *has-miR-181a* and increasing the expression of WIF-1 can inhibit tumor growth and metastasis (Ji et al., 2014). The EMT inhibitory effect of *miR-451* on hepatoma cells is mainly reflected in that *miR-451* targets the oncogene c-Myc to inhibit cell proliferation and reduces the activation of Erk1/2 signaling to control EMT-related marker proteins including E-cadherin and its MMPs family member (Huang JY et al., 2015). *miR-101* inhibits liver cancer cell proliferation and tumorigenicity by regulating the expression of SOX9, further research indicates that downregulation of *miR-101* promotes liver cell metastasis *in vitro* and associates poor prognosis of the patients with HCC (Zhang et al., 2012). The highly expressed hematopoietic-substrate-1-associated protein X-1 (HAX1) is targeted by *miR-125* in hepatoma cells. Overexpression of *miR-125* reduces the expression of mesenchymal phenotype-related genes including VEGF, N-cadherin, and vimentin, which results in inhibiting the proliferation and migration of hepatoma cells (Zha and Li, 2020). In addition, previous study shows that *miR-492*, *miR-519a*, *miR-21*, *miR-93*, and other regulatory factors are involved in the PTEN/AKT/GSK signaling pathway, and these regulatory factors promote cell proliferation and invasion by promoting cell proliferation and invasion to aggravate the EMT process of hepatoma cells (Bao et al., 2013; Jiang J et al., 2014; Ohta et al., 2015; Tu et al., 2016). Furthermore, the downregulation of *miR-204* under hypoxia-inducible conditions contributes to the upregulation of VASP at the post-transcriptional level. Studies have shown that overexpression of *miR-204* targets the abundance of regulated proteins under VASP inhibits the AKT/ERK signaling pathway, upregulates the E-cadherin gene, downregulates E-cadherin, Twist, MMPs family proteins, and reverses hypoxia induction. EMT process of liver cancer cells (Liu et al., 2018). Figure 3 shows that different miRNAs synergistically target and regulate the PTEN/PI3K/AKT signaling pathway to induce EMT development.

### 4.3 miRNA regulates the EMT process of hepatoma cells by the TGF-β signaling pathway

**FIGURE 4:**
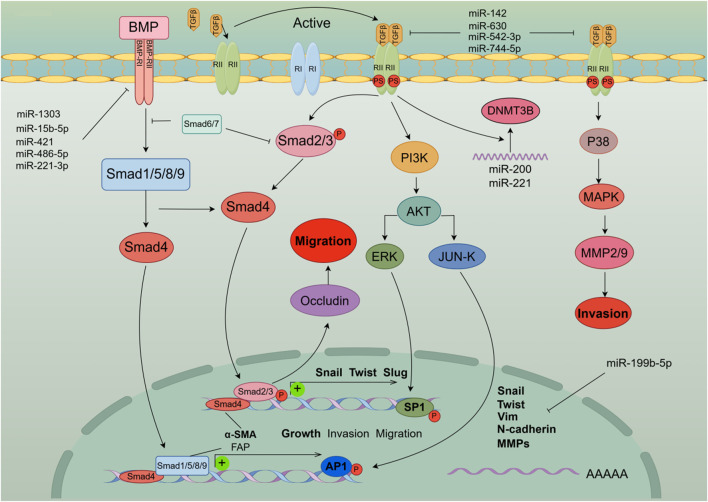
MiRNAs affect the occurrence and development of EMT in liver cancer cells by activating the TGF-β signaling pathway and cross-talking multiple signaling pathways including PI3 K and MAPK. In addition, the activation of the TGF-β signaling pathway initiates the expression of EMT-TFs genes and induces EMT in liver cancer cells.

The transforming growth factor (TGF)-β family is a multifunctional cytokine family that plays an important regulatory role in the development and pathogenesis of various diseases including fibrotic diseases, cardiovascular diseases, and cancer. there is a bidirectional regulatory relationship between miRNA and the TGF-β signaling pathway (Gregory et al., 2011). EMT is an orchestrated process by transforming growth factor (TGF)-β1, which is stored in ECM-rich tissue embedded in HCC cells (Giannelli et al., 2016). Previous studies have found that TGF-β activation regulates *miR-105*, *miR-199a*, *miR-215*, *miR-421*, *miR-529*, *miR- 21*, *miR-181*, *miR-155*, *miR-192*, and conversely, consistent with the diversity of miRNA target genes, computational prediction of miRNA targets suggests that multiple components of the TGF-β signaling pathway are targeted by multiple miRNAs This includes *miR-21*, *miR-17/92* cluster, *miR-106b*, *miR-211*, *miR-590* (Suzuki, 2018). In particular, the *miR-200* family consisting of TGF-β-downregulated miRNAs targets TGF-β, TβR-I, and Smad2 Thus, downregulation of the *miR-200* family by TGF-β enhances TGF-β signaling Conduction and induction of EMT (Gregory et al., 2011). In addition, *miR-15b-5p*, *miR-421*, *miR-1303*, *miR-221-3p*, and *miR-486-5p* are involved in TGF-β/BMP signaling pathway, angiogenesis, autophagy, and inflammation, promoting HCC malignant development and EMT transformation (Critelli et al., 2017). Hypermethylation is one of the factors of abnormal expression of miRNA, and also one of the signs of malignant transformation of tumors. Experiment data suggest that *miR-142* in tumor tissue is hypermethylated, which targets and regulates the expression of TGF-β and inhibits the activation of its signaling pathway. Low expression of *miR-142* accelerates the occurrence of EMT in hepatoma cells (Yu M. et al., 2017). *miR-200c* and *miR-34a* targeting EMT-related transcription factors Twist and Snail-1 have been reported in many studies to inhibit EMT development and regulate cell invasion and metastasis in hepatoma cells by activating TGF-β (Iwahashi et al., 2016). *miR-630* inhibits EMT by targeting Slug. Interestingly, the TGF-β-Erk/SP1 and JNK/c-Jun signaling pathways inhibit miR-630 by occupying transcription factor binding sites and thereby form a loop regulation, while Clinical data indicate that *miR-630*, as a tumor suppressor factor, is low expressed in patients with higher recurrence rate and shorter overall survival (Chen et al., 2016). Another study also showed that TGF-β activation could lead to *miR-374a-5p* targeting GADD45A via exosomes to promote EMT development (Lin et al., 2020a). Interestingly, it was mentioned above that *miR-542-3p* can play a role in the β-catenin signaling pathway, not only that, the existing experimental results show that *miR-542-3p* can directly target TGF-β1 to affect TGF-β/Smad signaling pathway, thereby inhibiting EMT development, which is consistent with clinical data that low expression of *miR-542-3p* is associated with poor prognosis (Zhang X. et al., 2018). previous research has established that *miR-199b-5p* binds to the 3′-UTR of N-cadherin mRNA, thereby reducing N-cadherin expression in HCC cells. Overexpression of *miR-199b-5p* inhibited TGF-b1-induced Akt phosphorylation, and inhibition of PI3K/Akt pathway blocked TGF-b1-induced N-cadherin expression to promote cell aggregation, inhibiting Cell migration and invasion in HCC cells and inhibition of tumor metastasis in nude mouse (Zhou et al., 2017). *miR-744-5p* targets TGF-β1 to inhibit this signaling pathway, thereby reversing EMT and suppressing HCC proliferation and invasion (Huang et al., 2021). Figure 4 shows the model diagram of miRNA regulating the TGF-β signaling pathway to affect the development of EMT in liver cancer cells.

### 4.4 miRNA regulates the EMT process of hepatoma cells through the RAS/MAPK signaling pathway

**FIGURE 5:**
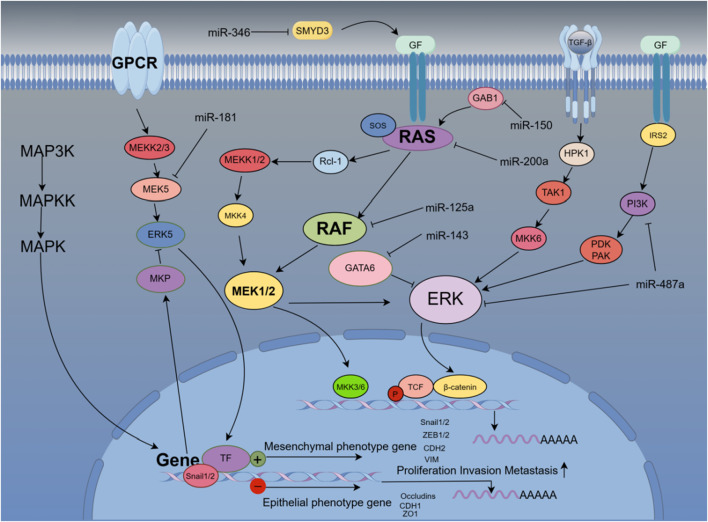
miRNAs accurately regulate the occurrence and development of EMT in liver cancer cells by targeting genes related to the Ras/MAPK pathway.

Mitogen-activated protein kinase (MAPK) pathways are characterized by their distinct kinase proteins and link extracellular signals to mechanisms that control physiological cellular processes such as growth, differentiation, proliferation, migration, and apoptosis. Alters in the MAPK cascade play critical roles in cancer development and progression (Dhillon et al., 2007). Different families of miRNA target and regulate the MAPK signaling pathway to affect the development of diseases, especially in liver cancer cells. A large number of studies have shown that miRNAs target this pathway protein to control the EMT process (Vasuri et al., 2018). *miR-346* targets the tumor-promoting factor SMYD3, which is highly expressed to activate MAP kinase signaling and promote the formation of Ras-driven cancers, resulting in the methylation of MAP3K2. Therefore, highly expressed *miR-346* can effectively inhibit liver cancer cells. proliferation and EMT transformation (Weiyou Zhu et al., 2017). NRG1 is a direct target of *miR-296-5p* and binds ERBB family members to activate downstream Janus kinase (JAK)/signal transducer and (STAT) transcriptional activators, phosphatidylinositol 3′-kinase (PI3K)-Akt and MAPK, which leads to cell proliferation, invasion, and angiogenesis, and promotes EMT. Highly expressed *miR-296* reverses the proliferation and invasion of hepatoma cells (Shi et al., 2018). The hypermethylation of *miR-129-3p* promoter in metastatic tissues may be one of the inducements for hypermetastatic HCC. Mechanistically, *miR-129-3p* targeting Aurora-A, as a serine/threonine protein kinase, activates PI3K/Akt and p38-MAPK signaling to promote EMT, invasion *in vitro* and metastasis *in vivo* in HCC cells (Shiyun Cui et al., 2016). *miR-181* directly regulates MAPK phosphatase-5 (MKP-5) to inhibit the phosphorylation of downstream p38 MAPK to block polycyclic aromatic hydrocarbons (PAH)-induced hepatocellular carcinoma cell invasion and migration (Song et al., 2013). The low expression of *miR-143* in liver cancer patients targets the regulation transcription factor GATA-binding factor 6 (GATA6) to inhibit the growth and invasion of liver cancer cells (Xue et al., 2017). miR-125a targets MMP11, SIRT7, and c-Raf to regulate MAPK signaling and inhibit hepatocellular carcinoma cell proliferation (Coppola et al., 2017). As an activator of RAS, GAB1 is highly expressed in HCC patients. Studies have found that miR-200a targets and binds to GAB1 to inhibit RAS/MAPK signaling, thereby reducing the migration and invasion ability of liver cancer cells (Wang et al., 2017). Similarly, high levels of *miR-150* reduced GAB1 expression, subsequently downregulated phosphorylated ERK1/2 and inhibited EMT, while low levels of *miR-150* were associated with worse outcomes in HCC patients. Clinicopathological features are significantly associated with poor prognosis (Wei Sun et al., 2016). Experimental results demonstrate that upregulated *miR-487a* correlates with a poor prognosis for HCC patients. *miR-487a* directly binds the germination-associated EVH1 domain containing 2 (SPRED2) or phosphoinositide-3-Kinase regulatory subunit 1 (PIK3R1) to enhance HCC cell proliferation and metastasis. Importantly, *miR-487a* promotes MAPK signaling through SPRED2 and promotes proliferation via PIK3R1-mediated AKT signaling (Chang R.M. et al., 2017). *miR-188-5p* relies on MAPK signaling to inhibit HCC cell proliferation and metastasis by targeting fibroblast growth factor 5 (FGF5) (Fang et al., 2015). Figure 5 shows that miRNAs affect the EMT process of liver cancer cells through multi-target regulation of the RAS/MAPK signaling pathway.

All the miRNAs involved in this part depend on different signaling pathways to regulate biological functions summarized in Table 1.

**TABLE 1:** miRNAs depend on various signaling pathways to perform biological functions.

miRNA	Pathway	Direct target	Function	Reference
miR-1088	Wnt/β-catenin Pathway	FOXO1	promote EMT	Lin et al., 2019
miR-197		Axin-2 NKD1 DKK2	promote EMT	Hu et al., 2018
miR-498		ZEB2	inhibit EMT	Zhang X et al. 2018
miR-542-3P		FZD7	inhibit EMT	Wu et al. 2017
miR-1246		RORα	promote EMT	Huang et al., 2020
miR-504		FZD7	inhibit EMT	Quan et al., 2018
miR-298		CTNND1	inhibit EMT	Cao N et al., 2018
miR-708		ZEB1	reverse EMT	Yang B et al., 2020
miR-122		Snail1 Snail2	inhibit EMT	Jin et al., 2017
miR-541-5p		ZIC1	promote EMT	Li D et al., 2021
miR-219-5p		GPC3	inhibit EMT	Gong et al., 2019
miR-194		PRC1	inhibit EMT	Zhang et al., 2019
miR-25-3p	PTEN/PI3K/Akt Pathway	PTEN	promote EMT	Wang et al., 2020
miR-130b-3p		PTEN	promote EMT	Wang et al., 2020
miR-425-5p		PTEN	promote EMT	Wang et al., 2020, Fang et al., 2017
miR-345		IRF1	inhibit EMT	Yu Q et al., 2017
miR-148a		Met	inhibit EMT	Zhang et al., 2014
miR-21		HIF-1α HIF-2α PDK1	promote EMT	Tian et al., 2019
miR-10b		PTEN	promote EMT	Tian et al., 2019
miR-150-3p		PTEN	inhibit EMT	Yugawa et al., 2021
miR-155-5p		PTEN	promote EMT	Fu et al., 2017; Sun et al., 2019
miR-32-5p		PTEN	promote EMT	Fu et al., 2018;
miR-92a-3p		PTEN	promote EMT	Yang J et al., 2020
miR-181a		WIF-1	promote EMT	Ji et al., 2014
miR-451		c-Myc	inhibit EMT	Huang J Y et al., 2015
miR-101		SOX9	inhibit EMT	Zhang et al., 2012
miR-125		HAX1	inhibit EMT	Zha and Li, 2020
miR-492		PTEN	promote EMT	Bao et al., 2013
miR-519A		PTEN	promote EMT	Jiang J et al., 2014

miRNA	Pathway	Direct target	Function	Reference
miR-21	PTEN/PI3K/Akt Pathway	PTEN	promote EMT	Ohta et al., 2015
miR-93		PTEN	promote EMT	Tu et al., 2016
miR-204		VASP	inhibit EMT	Liu et al., 2018
miR-129-3p		Aurara-A	promote EMT	Shiyun Cui et al., 2016
miR-21	TGF-β Signaling	TGF-β	inhibit EMT	Suzuki, 2018
miR-17/92		TGF-β	inhibit EMT	Suzuki, 2018
miR-106b		TGF-β	inhibit EMT	Suzuki, 2018
miR-211		TGF-β	inhibit EMT	Suzuki, 2018
miR-590		TGF-β	inhibit EMT	Suzuki, 2018
miR-200		TGF-β TβR-I Smad2	inhibit EMT	Gregory et al., 2011
miR-15b-5p		BMP	inhibit EMT	Critelli et al., 2017
miR-421		BMP	inhibit EMT	Critelli et al., 2017
miR-1303		BMP	inhibit EMT	Critelli et al., 2017
miR-221-3p		BMP	inhibit EMT	Critelli et al., 2017
miR-486-5p		BMP	inhibit EMT	Critelli et al., 2017
miR-142		TGF-β	inhibit EMT	Yu M et al., 2017
miR-200c		Twist, Snail-1	inhibit EMT	Iwahashi et al., 2016
miR-34a		Twist, Snail-1	inhibit EMT	Iwahashi et al., 2016
miR-630		Slug	inhibit EMT	Chen et al., 2016
miR-374-5p		GADD45A	promote EMT	Lin et al., 2020a
miR-542-3p		TGF-β1	inhibit EMT	Zhang X et al., 2018
miR-199b-5p		CDH2	promote EMT	Zhou et al., 2017
miR-744-5p		TGF-β1	inhibit EMT	Huang et al., 2021
miR-346	RAS/MAPK signaling pathway	SMYD3	inhibit EMT	Weiyou Zhu et al., 2017
miR-296-5p		NRG1	promote EMT	Shi et al., 2018
miR-296		ERBB	inhibit EMT	Shi et al., 2018
miR-129-3p		Aurara-A	promote EMT	Shiyun Cui et al., 2016
miR-181		MKP-5	inhibit EMT	Song et al., 2013
miR-143		GATA6	promote EMT	Xue et al., 2017
miR-125a		MMP11, SIRT7, and c-Raf	inhibit EMT	Coppola et al., 2017
miR-200a		GAB1	inhibit EMT	Wang et al., 2017

miRNA	Pathway	Direct target	Function	Reference
miR-150	RAS/MAPK signaling pathway	GAB1	inhibit EMT	Wei Sun et al., 2016
miR-487a		SPRED2 PI3KR1	inhibit EMT	Chang R M et al., 2017
miR-188-5p		FGF5	inhibit EMT	Fang et al., 2015

## 5 miRNAs regulate EMT independently of the aforementioned Signaling pathways

Exosomes are important carriers of confidence transmission among T cells, B cells, dendritic cells, epithelial cells, and tumor cells, and their size ranges from 30 to 100 nm in diameter. Multiple studies have demonstrated that abundant cell-free exosome-derived miRNAs can induce miRNA-mediated target gene repression and function effectively as oncogenes or tumor suppressors in various pathological processes in recipient normal or tumor cells (Valadi et al., 2007; Taylor and Gercel-Taylor, 2008). There is emerging evidence indicating that exosomes play a central role in cell-to-cell communication. Existing studies have shown that dysregulation of exosomal microRNAs (miRNAs), including *miR-125b-5p*, *miR-374a-5p, miR-24-3p*, *miR-200b-3p*, and *miR-21-5p*, play an important role in the progression of hepatocarcinogenesis (Lin et al., 2020b).

The study has found that the expression of *miR-144-3p* in patients with advanced liver cancer was significantly reduced, further evidence indicates that *miR-144-3p* targets the regulation of endogenous bornavirus-like nucleoprotein EBLN3P and dedicator of cytokinesis 4 DOCK4 to inhibit the proliferation and invasion of liver cancer cells. Interestingly, an in-depth study of the mechanism by which EBLN3P competes with *miR-144-3p* to reduce the latter’s inhibitory effect on DOCK4 promotes EMT transition in HCC cells. Therefore, inhibiting the expression of *miR-144-3p* and increasing EBLN3P played a similar promoting effect, while overexpression of *miR-144-3p* and reducing DOCK4 exerted a similar inhibitory effect on EMT (Li et al., 2020). Another research found that low expression of *miR-124* promotes cell migration, and overexpression of *miR-124* can directly target Rho-associated coiled-coil and (EZH2) genes containing protein kinase 2 (ROCK2) to destabilize its mRNA and inhibit EMT (Zheng et al., 2012). *miR-205* targeting axon guidance factor 4C (SEMA4C) promotes apoptosis, inhibits proliferation, and attenuates tumor cell EMT In liver cancer cells (Lu et al., 2018). In contrast, expression of miR-194 in liver stromal cells leads to decreased levels of the mesenchymal phenotype gene N-cadherin, preventing cell migration and invasion (Meng et al., 2010). *miR-532-3p* targets and regulates kinesin family member C1 (KIFC1) a tumor-promoting factor, which regulates HCC cell metastasis and invasion through Gankyrin-dependent activation of Twist (Han et al., 2019). Low expression of *miR-34* leads to poor prognosis and enhanced hepatocyte migration and invasion *in vitro* in HCC patients (Zhang et al., 2019; Silveira et al., 2022). Similarly, *miR-449a* can also target the regulation of Met protein abundance, reduce the accumulation of Snail in the nucleus, reduce the level of mesenchymal markers, and increase the expression of epithelial markers, ultimately blocking the EMT of liver cancer cells. Aberrantly expressed *miR-449a* is often accompanied by high metastasis and enhanced the invasive ability of liver cancer cells (Chen et al., 2015). Activation of p53 promoted the expression of *miR-200* and *miR-192*, which inhibited the expression of ZEB1/2 and blocked the EMT transition of hepatoma cells (Kim et al., 2011). Previous clinical studies clarified that the prognosis of patients with low expression of *miR-26a-5p* was significantly worse than that of patients with high expression, and a strong downregulation of *miR-26a-5p* was observed in tumor tissue, research suggests that low expression of *miR-26a-5p* upregulated vimentin expression and downregulated E-cadherin expression (Chang L. et al., 2017). *miR-519c-3p* promotes HCC cell proliferation, migration, and invasion *in vitro* and enhances HCC cell growth and metastasis *in vivo*. Mechanistically, B cell translocation gene 3 (BTG3) was identified as a direct downstream target of *miR-519c-3p*, which acts as a tumor promoter in regulating HCC growth and metastasis by targeting BTG3 and may become a new therapeutic target for HCC (Wang et al., 2019). *miR-301b-3p* directly binds to the 3′UTR of degenerate-like family member 4 (VGLL4) and negatively regulates its expression. Meanwhile, high *miR-301b-3p* levels predict a significant decrease in overall survival in HCC patients. Knockdown of *miR-301b-3p* inhibits cell proliferation, causes cell cycle arrest in the G2/M phase, and induces apoptosis in Huh7 and Hep3B cells, inhibiting cell invasion and migration, thereby blocking EMT progression (Guo et al., 2019). *miR-5692a* has an oncogenic effect in HCC by targeting HOXD8, and reducing *miR-5692a* can effectively improve the proliferation and invasion of HCC cells and promote cell apoptosis (Shijie Sun et al., 2019). *miR-455* normally acts as a tumor suppressor in various cancers, and in liver cancer cells, *miR-455* inhibits cell viability and invasion by directly targeting RAB18. Increasing *miR-455* expression is expected to improve patient prognosis (Wang et al., 2021). The regulation of EMT by miRNA targeting Twist-1 has been well reported in numerous works of literature (Feng et al., 2022). Interestingly, Twist-1 can also regulate miRNA to form miRNA/Twist-1/miRNA loop regulation, and the upregulation of Twist-1 leads to 18 differential expressions of miRNAs, among which *miR-27a-3p* downregulation clamps the EMT process of hepatoma cells (Zhao et al., 2016). As an activator of EMT, ZEB1 is a member of the zinc finger family, encoding zinc finger and homeodomain transcriptional cytokines (Li L. Y. et al., 2021). It has been widely reported that miRNAs controlling ZEB1 can reduce the proliferation and invasion of hepatoma cells to block EMT (Xu et al., 2018; Feng et al., 2022).

Of note, an increasing number of studies have shown that miRNAs enable control of cellular EMT processes through cellular metabolism. On the one hand, *miR-612* reduces HCC pseudopodia formation and inhibits metastasis and invasion through HADHA-mediated lipid reprogramming, which is consistent with clinical data that patients with low *miR-612* levels and high HADHA levels have a poorer prognosis and shorter overall survival (Liu et al., 2020). In terms of glucose metabolism, the Limb and CNS expressed 1-like (LIX1L) regulates *miR-21-3p* expression to target fructose-1,6-bisphosphatase (FBP1). The study found that LIX1L promotes *miR-21-3p* expression to inhibit FBP1 protein abundance, resulting in attenuated gluconeogenesis, thereby promoting EMT development. Conversely, knockdown of LIX1L and inhibition of *miR-21-3p* expression can block the transformation of hepatocytes into carcinogenesis. and reverse the EMT process (Zou et al., 2021). In addition, numerous reports have demonstrated that miRNAs also play critical roles in metabolic homeostasis, and *miR-122* plays a central role in maintaining liver homeostasis by affecting various genes involved in hepatic cholesterol and lipid metabolism (Szabo and Bala, 2013). The biological functions of miRNAs independent of signaling pathways are summarized in Table 2.

**TABLE 2:** miRNAs play biological functions independent of signaling pathways.

miRNA	Direct target	Function	Reference
miR-144-3p	EBLN3P DOCK4	inhibit EMT	Li et al., 2020
miR-124	ROCK2,E2H2	inhibit EMT	Wang et al., 2019
miR-205	SEMA4C	inhibit EMT	Lu et al., 2018
miR-194	CHD2	inhibit EMT	Meng et al., 2010
miR-532-3p	KIFC1	promote EMT	Han et al., 2019
miR-34	SNAIL1	inhibit EMT	Zhang et al., 2019; Silveira et al., 2022
miR-449a	Met	inhibit EMT	Chen et al., 2015
miR-200	ZEB1/2	inhibit EMT	Kim et al., 2011
miR-192	ZEB1/2	inhibit EMT	Kim et al., 2011
miR-26a-5p	CDH2	inhibit EMT	Chang L. et al., 2017
miR-519c-3p	BTG3	promote EMT	Wang et al., 2019
miR-301b-3p	VGLL4	inhibit EMT	Guo et al., 2019
miR-5692a	HOXD8	promote EMT	Shijie Sun et al., 2019
miR-455	RAB18	inhibit EMT	Wang et al., 2021
miR-27a-3p	Twist-1	inhibit EMT	Zhao et al., 2016
miR-612	HADHA	inhibit EMT	Liu et al., 2020
miR-21-3p	FBP1	promote EMT	Zou et al., 2021

## 6 Developing promising therapeutic strategies for miRNA

The application of microRNA in cancer treatment is a new and exciting method to interfere with the molecular mechanism of malignant tumors. There are several major advantages for the clinical application of miRNA, the first is as a biomarker for cancer prediction and diagnosis, easy to detect and sample (Volinia S et al., 2006). Secondly, miRNAs have a wide range of miRNA, according to statistics, more than 30% of the genes can accept the regulation from miRNA (Corsini et al., 2012). In addition, multi-site, multi-target artificial miRNA development strategies can be designed for specific tumors (Wang, 2011). However, clone selection is laborious and targeting miRNAs generated from multiple loci remains a challenge (Lu and Rothenberg, 2018). To provide a more reference and promising research direction, and to better serve clinical practice, multi-treatment strategies have been accurately proposed in the field of non-coding RNA. Firstly, miRNA has been used as a biomarker in various studies to promote the identification and characterization of tumorigenesis, which can rapidly diagnose diseases in clinical diagnosis (Acunzo et al., 2015). Five strategies have been proposed for targeting endogenous oncogenic miRNAs including knocking down *in vivo* expression, development of antisense oligonucleotide strands for miRNAs, development of inhibitors, development of miRNA sponges such as long non-coding RNAs and circular non-coding RNAs, and depletion-type single-stranded fragments developed for target gene mRNA sequences (Qadir MI, 2017). In particular, miRNA oligomers are subdivided into anti-miRNA oligonucleotides (AMOs) and lock-nucleic acid antisense oligonucleotides (lock-nucleic acid antisense oligonucleotides, LNAs), miRNA sponges, miRNA masks, nanoparticles, antagonists, and multi-target antisense miRNA oligonucleotides (MTg-AMOs). Anti-miRNA-221 developed for *miRNA-221* has a significant inhibitory effect on HCC and improved survival, and clinical trials have also obtained encouraging results (Bae et al., 2015). In addition, based on the reintroduction of single or multiple mimetic micro-RNA (non-natural double-stranded micro-RNA homologous RNA fragments) into a group of tumor cells, attempts are made to reconstruct the endogenous normal expression profile and restore or lose function. They include gene therapy, miRNA mimics, siRNAs miRNA, antagonists, and small-molecule inhibitors (Bader et al., 2011). Based on the tolerance of cancer cells to miRNA participation in a special period, the development of interventions targeting multiple miRNA mimics may inhibit multiple signaling pathways, thereby improving the efficacy of miRNA mimics to achieve satisfactory results (Li and Lu, 2014). Previous studies have shown that oncolytic viruses have been shown to interact with miRNA expression in HCC. Such carriers have tumor-specific effects and can cause cytolysis of tumor cells; therefore, healthy tissues have low toxicity *in vivo*. Therefore, oncogenic viruses can be used as an optimization strategy for cancer treatment (Slaney and Darcy, 2015). Regulatory miRNA sequences are inserted into the genome of conditionally replicating adenoviruses (CRAds) to enable the liver to specifically inhibit viral replication. In addition, oncolytic viruses have also been used in HCC xenotransplantation models to develop let-7-dependent adenoviruses, which can only replicate in cancer cells and not in normal healthy liver tissue. miRNA-199-dependent CRAd has also been developed, which can replicate in liver cancer cells but is less common in normal healthy liver tissue (Ulasov et al., 2013).

Notably, although nucleic acid drugs such as miRNAs have targeted regulatory effects, however, similar to other therapeutic oligonucleotides, the main challenge remains the successful delivery of therapeutic miRNAs to target tissues without compromising miRNA integrity, because naked ribonucleic acid is subject to nuclease-dependent rapid degradation and is therefore unstable in the liquid environment of the organism (Davis et al., 2008; Whitehead et al., 2009; Kaasgaard T 2010). Therefore, many therapeutic applications of RNAi are limited to local administration and limit the exposure of RNAi reagents to potential degradation mechanisms to a minimum (Akhtar and Benter, 2007). However, topical administration is only applicable to a limited local area of the target tissue and is generally not conducive to the exposure of all diseased cells to the drug. Therefore, systemic administration is a better route of administration, and the development and exploration of miRNA drug delivery systems is also an important part of miRNA targeted therapy (Bader et al., 2011). Another advantage of the in-depth development of miRNA targeted therapy is that it will be used as an emerging means of preclinical diagnosis (He et al., 2020). A typical example is the upregulation of *miR-221* in liver cancer cells whichhas been confirmed by multiple studies as a potential biomarker for the detection of hepatocellular carcinoma (Karakatsanis et al., 2011; Yasser et al., 2021; Di Martino et al., 2022). More importantly, as an innovative diagnostic indicator, miRNA is usually obtained from gastric juice, urine, saliva, and blood as a non-invasive detection method, which greatly increases its application value (Baraniskin et al., 2011; Vila-Navarro et al., 2017; Cao Y. et al., 2018). The high-sensitivity detection technology of *miR-21* in breast cancer developed with the sensing effect of nanomaterials has promoted the early diagnosis of breast cancer (Salahandish et al., 2018). Equally exciting is the use of Nano-String technology, or nCounter technology, which is a quantitative method without digital amplification, to identify the content of MicroRNA-1246 in the dysregulated serum of prostate cancer, to facilitate clinical diagnosis (Bhagirath et al., 2018).

Therapeutic miRNAs constitute a special form of targeted therapy, and tools and diagnoses that have been promoted for personalized medicine and used to accurately characterize cancer genotypes in patients will become valuable resources for the implementation of miRNA-based treatments in diseases (Weidhaas et al., 2007). In addition, miRNA-based therapies have strong anti-cancer activity in animal models, and therapeutic miRNAs may fulfill their promise by potentially safely and unimpeded translation of miRNA replacements into clinical practice. The recent role of miRNAs in cancer stem cells, and their ability to make cancer cells more sensitive to radiation and conventional chemotherapy, will create new opportunities for more effective treatment of cancer (Liu et al., 2011; Lai et al., 2018; Luo J et al., 2019).

Finally, we are also concerned that miRNA therapy can be combined with chemotherapy, radiotherapy, and immunotherapy, to providing new methods for miRNA-based therapy and the use of alternative therapies and miRNA mimics. Meanwhile, miRNA, together with other ncRNAs such as lncRNA and circRNA, has important clinical significance in the pathogenesis, diagnosis, and treatment of human cancer. More relevant studies should be valued by researchers to fully understand the pathogenesis of cancer and improve targeted therapy.

## 7 Conclusion and perspectives

Based on the complex biological background and elusive regulatory network of HCC, in-depth exploration of its molecular pathogenesis requires a systematic understanding of the body’s system solution strategy between various external stresses and internal mutations. miRNAs, as a crucial part of the complex regulatory network in organisms, play a pivotal role in post-transcriptional regulation. As an essential biological process in cancer metastasis and invasion, EMT is systematically regulated by miRNAs, and this system is reflected in the interactive dialogue of multiple signaling pathways. Distinct pathways have predominant roles in the initiation and progression of EMT and regulate changes in morphology and gene expression that convert epithelial cells into motile mesenchymal cells for hepatocellular carcinoma initiation and progression. Identification of the background in pharmacy, molecular biology, and cell biology will identify the cross-regulation of multiple signaling pathways *in vivo*. In addition, a comprehensive and objective understanding of the EMT-mediated miRNA regulatory network in the process of hepatocellular carcinogenesis is imminent to provide new insights for further mechanism research and clinical drug development and screening. Further efforts need to focus on clinical development, especially in the areas of miRNA family drug resistance and tumor-targeted therapy, according to these discovered mechanisms. If all these efforts are completed, new treatment strategies for advanced hepatocellular carcinoma will be determined, and better methods for outcome prediction will be provided.

## Figures and Tables

**Table:** 

**HCC**	Hepatocellular carcinoma
**miRNAs**	MicroRNAs
**EMT**	Epithelial-Mesenchymal Transition
**PTEN**	Phosphatase and tensin homology deleted on chromosome ten
**PI3K**	Phosphatidylinositide 3-kinases
**AKT**	Protein kinase B
**TGF-β**	Transforming growth factor-β
**RAS**	Rat sarcoma
**MAPK**	Mitogen-activated protein kinase
**HBV**	Hepatitis B
**HCV**	Hepatitis C
**UTR**	Untranslated region
**MMPs**	Matrix metallopeptidase family proteins
**GRB2**	Growth factor receptor binding protein 2
**EGF**	Epidermal growth factor
**FGF**	Fibroblast growth factor
**HGF**	Hepatocyte growth factor
**VEGF**	Vascular endothelial growth factor
**RTKs**	Receptor tyrosine kinases
**GSK3β**	Glycogen synthase kinase-3
**LEF**	Lymphoid enhancer-binding factor 1
**TCF**	T cell factor
**HIF1α**	Hypoxia-inducible factor 1α
**FOXO1**	Forkhead Box O1
**NKD1**	Naked Cuticle 1
**DKK2**	Dickkopf-related protein 2
**ZEB2**	Zinc Finger E-Box Binding Homeobox 2
**FZD7**	Frizzled Class Receptor 7
**RORα**	RAR Related Orphan Receptor A
**CTNND1**	Catenin Delta 1
**WNT11**	Wnt Family Member 11
**MMP7**	Matrix Metallopeptidase 7
**HSCs**	Hepatic Stellate Cells
**ZIC1**	Zic Family Member 1
**MMP2**	Matrix Metallopeptidase 2
**GPC3**	Glypican-3
**PRC1**	Protein Regulator Of Cytokinesis 1
**CXCL12**	C-X-C Motif Chemokine Ligand 12
**CXCR4**	C-X-C Motif Chemokine Receptor 4
**CAFs**	Cancer-associated fibroblasts
**PDK1**	Pyruvate Dehydrogenase Kinase 1
**MMP9**	Matrix Metallopeptidase 9
**bFGF**	Fibroblast Growth Factor 2
**SCAI**	Suppressor Of Cancer Cell Invasion
**β1-Fak**	β1 integrin-focal adhesion kinase
**CDC42**	Cell Division Cycle 42
**TIMP2**	TIMP Metallopeptidase Inhibitor 2
**mTOR**	Mechanistic Target Of Rapamycin Kinase
**STAT3**	Signal Transducer And Activator Of Transcription 3
**Met**	Tyrosine-Protein Kinase Met
**GSK-3β**	Glycogen Synthase Kinase 3 Beta
**TME**	Tumor microenvironment
**HIF-1α**	Hypoxia-Inducible Factor 1-Alpha
**HIF-2α**	Hypoxia-Inducible Factor 2 Alpha
**HRE**	Hypoxia-response element
**WIF-1**	WNT Inhibitory Factor 1
**c-Myc**	MYC Proto-Oncogene, BHLH Transcription Factor
**Erk**	extracellular regulated protein kinases
**SOX9**	SRY-Box Transcription Factor 9
**HAX1**	Hematopoietic-substrate-1-associated protein X-1
**VASP**	Vasodilator Stimulated Phosphoprotein
**ECM**	Extracellular matrix
**BMP**	Bone Morphogenetic Protein
**SP1**	Sp1 Transcription Factor
**JNK**	c-Jun N-terminal kinase
**GADD45A**	Growth Arrest And DNA Damage Inducible Alpha
**TGF-b1**	Transforming Growth Factor Beta 1
**SMYD3**	SET And MYND Domain Containing 3
**MAP3K2**	Mitogen-Activated Protein Kinase Kinase Kinase 2
**NRG1**	Neuregulin 1
**ERBB**	Erb-B2 Receptor Tyrosine Kinase
**MKP-5**	MAPK phosphatase-5
**PAH**	Polycyclic aromatic hydrocarbons
**GATA6**	GATA-binding factor 6
**MMP11**	Matrix Metallopeptidase 11
**SIRT7**	Sirtuin 7
**c-Raf**	Raf-1 Proto-Oncogene, Serine/Threonine Kinase
**GAB1**	GRB2 Associated Binding Protein 1
**SPRED2**	Germination-associated EVH1 domain containing 2
**PIK3R1**	Phosphoinositide-3-Kinase regulatory subunit 1
**FGF5**	Fibroblast growth factor 5
**EBLN3P**	Endogenous bornavirus-like nucleoprotein 3
**DOCK4**	Dedicator of cytokinesis 4
**ROCK2**	Rho-associated coiled-coil containing protein kinase 2
**SEMA4C**	Semaphorin 4C
**KIFC1**	Kinesin Family Member C1
**BTG3**	B cell translocation gene 3
**VGLL4**	Vestigial Like Family Member 4
**HOXD8**	Homeobox D8
**RAB18**	RAB18, Member RAS Oncogene Family
**Twist-1**	Twist Family BHLH Transcription Factor 1
**ZEB1**	Zinc Finger E-Box Binding Homeobox 1
**HADHA**	Hydroxyacyl-CoA Dehydrogenase Trifunctional Multienzyme Complex Subunit Alpha
**LIX1L**	Limb and CNS expressed 1 like
**FBP1**	Fructose-1,6-bisphosphatase
**MTg-AMOs**	multi-target antisense miRNA oligonucleotides
**CRAds**	conditionally replicating adenoviruses
